# Two conformationally distinct α-synuclein oligomers share common epitopes and the ability to impair long-term potentiation

**DOI:** 10.1371/journal.pone.0213663

**Published:** 2019-03-22

**Authors:** Femke van Diggelen, Dean Hrle, Mihaela Apetri, Gunna Christiansen, Gerhard Rammes, Armand Tepper, Daniel Erik Otzen

**Affiliations:** 1 Interdisciplinary Nanoscience Center (iNANO), Aarhus University, Aarhus C, Denmark; 2 Crossbeta Biosciences BV, Utrecht, The Netherlands; 3 Klinik für Anaesthesiologie der Technischen Universität München, Klinikum Rechts der Isar, Munich, Germany; 4 Department of Biomedicine, Aarhus University, Aarhus C, Denmark; INRA Centre de Jouy-en-Josas, FRANCE

## Abstract

Parkinson’s Disease (PD) is a neurodegenerative disease for which there currently is no cure. Aggregation of the pre-synaptic protein α-synuclein (aSN) into oligomers (αSOs) is believed to play a key role in PD pathology, but little is known about αSO formation *in vivo* and how they induce neurodegeneration. Both the naturally occurring polyunsaturated fatty acid docosahexaenoic acid (DHA) and the lipid peroxidation product 4-hydroxynonenal (HNE), strongly upregulated during ROS conditions, stimulate the formation of αSOs, highlighting a potential role in PD. Yet, insight into αSOs structure and biological effects is still limited as most oligomer preparations studied to date are heterogeneous in composition. Here we have aggregated aSN in the presence of HNE and DHA and purified the αSOs using size exclusion chromatography. Both compounds stimulate formation of spherical αSOs containing anti-parallel β-sheet structure which have the same shape as unmodified αSOs though ca. 2-fold larger. Furthermore, the yield and stabilities of these oligomers are significantly higher than for unmodified aSN. Both modified and unmodified αSOs permeabilize synthetic vesicles, show high co-localisation with glutamatergic synapses and decrease Long Term Potentiation (LTP), in line with the reported synaptotoxic effects of αSOs. We conclude that DHA- and HNE-αSOs are convenient models for pathogenic disease-associated αSOs in PD.

## Introduction

Neurodegenerative diseases, such as Parkinson’s disease (PD), typically involve aggregation of proteins into amyloid fibrils.[1] In PD these intra-neuronal deposits are called Lewy Bodies, and consist mainly of the protein α-synuclein (aSN). Aggregation of aSN into fibrils[2,3] is an important hallmark of PD, but rather than the fibrils themselves, aggregation intermediates called aSN oligomers (αSOs) are believed to be the cytotoxic species.[4] In support of this, toxicity in both cellular[5] and animal models[6] is often observed without large aSN aggregates. Moreover, neurodegeneration is highest in animal models expressing E35K and E57K aSN mutations, which show increased αSO formation, but is lowest when expressing the A53T mutant, associated with increased fibril-formation.[4]

Despite their importance, the structures and mode of toxicity of αSOs remain unclear. Toxicity appears to involve membrane permeabilization (plasma-, mitochondrial, ER, vesicle membrane) and promiscuous protein binding (membrane receptors, cytosolic-, synaptic proteins).[7] More specifically, αSOs induce increased Ca^2+^ influx[8] and reactive oxygen species (ROS) production[9] and induce synaptotoxicity in the form of decreased neuron excitability,[10] increased membrane conductance,[8] decreased synaptic firing[11] and impaired long-term potentiation (LTP).[12] αSOs detected *in vivo* are protein K resistant,[13] FILA-1 positive[14] and lipid dependent[15] However, brain-derived αSOs can only be extracted from patients post-mortem when the disease is already well advanced, and extraction methods can alter or disrupt the oligomers. Most knowledge of αSO structure and toxicity therefore derives from *in vitro* studies, where αSOs are generated upon monomer overexpression in cells, or monomer aging in cell-free systems. Many different types of αSOs have been generated *in vitro* under different conditions, with shapes ranging from spherical to annular and chain-like.[16] This diversity makes it difficult to form a single unifying model of how αSOs are formed, their relationship to fibrillation proper, which αSOs are disease-relevant and what their molecular structures are.[17] Nevertheless, most groups report spherical αSOs[16] consisting of around 30 monomers.[17,18] There is broad agreement that the αSOs contain a rigid (anti-parallel) β-sheet core comprising a central region of aSN, surrounded by a flexible unordered layer comprising the C-terminus.[19]

αSO formation can be stimulated by the addition of lipids such as polyunsaturated fatty acids (PUFAs).[20] Both long-chain PUFAs[21] and αSOs[15] occur at elevated levels in several brain regions of PD and dementia with Lewy Bodies (DLB) patients. One of the most abundant PUFAs in the brain is the 22-carbon docosahexaenoic acid (DHA)[22] which is mainly localised at synapses.[23] DHA is mainly esterified to lipids such as triacylglycerol or phospholipids, but it can be released, amongst others, by oxidation in response to ischemia, excitotoxicity, inflammation, cholinergic- or serotonergic receptor stimulation.[22] DHA’s six non-conjugated double bonds make it highly susceptible to oxidation by ROS,[24] light, Cu^2+^, Fe^2+^ or molecular oxygen.[25] A series of peroxidation and decomposition steps lead to products such as the reactive aldehyde 4-hydroxyhexenal (HHE), which can form Schiff base- and Michael adducts with Cys, His and Lys residues.[26] DHA and its breakdown products induce spherical and annular αSOs which do not aggregate further into mature fibrils[20] but permeabilize synthetic vesicles and modestly increase the permeability of SH-SY5Y cells to otherwise cell-impermeable propidium iodide.[27] However, we have limited knowledge of the structural and functional properties of these DHA-αSOs species. Furthermore, most studies have used aggregation mixtures, which contain different aggregated and modified species of aSN.[16]

Peroxidation of the PUFA arachidonic acid also results in the release of smaller molecular fragments, such as the highly reactive molecule 4-hydroxynonenal (HNE),[26] which covalently modifies proteins and DNA.[26] Oxidative stress can increase HNE concentrations from the normal 0.1–3 μM range up to 10–5000 μM.[26] HNE can undergo Michael addiction with its electrophilic C3 atom, and a Schiff base formation with its carbonyl group, allowing it to form both intra- and intermolecular crosslinks.[28] There is conflicting evidence on HNE’s ability to crosslink aSN[29] but HNE modification of aSN is undisputed. 58% of nigral neurons in PD patients contain HNE-modified proteins, in contrast to only 9% in age matched healthy controls.[30] Like DHA, HNE stimulates the formation of αSOs[29,31] with shapes ranging from spherical, curvilinear, ring-like, globular to protofibrillar and with either β-sheet or random coil structures.[16] HNE-αSOs are cytotoxic against SH-SY5Y cells and primary neurons,[31,32] and stimulate ROS production *in vitro*.[32] Extracellular HNE-αSOs are proposed to contribute to pathology by inducing synaptotoxicity, supported by their ability to bind to primary neurons, invade SH-SY5Y cells, and impair LTP, most likely involving NMDA receptor activation.[29,31,33]

Overall it is unclear which oligomeric (sub)species is responsible for which experimentally observed effect as most oligomer preparations studied to date are heterogeneous in composition. In an attempt to clarify this, we have generated and isolated αSOs in the presence of DHA or HNE (which we refer to as DHA-αSOs and HNE–αSOs) and compared them to αSOs made in buffer without additional components (unmodified αSOs). Oligomers were purified by size exclusion chromatography (SEC), followed by a detailed structural analysis using a range of techniques. We show that despite the clear chemical and biophysical differences, all three classes of αSOs are spherical oligomers which co-localize with excitatory synapses and decrease LTP, suggesting a common toxic mechanism based on the same overall structure.

## Materials and methods

### Preparation of monomeric aSN

WT human aSN was recombinantly expressed in *E*. *coli*, purified as described,[2,34] lyophilized and stored at -20°C. Concentrations were determined using UV-Vis absorbance (Spectramax plus 384, Molecular Devices, Sunnyvale, CA) in a 1 cm quartz cuvette using a calculated extinction coefficient of 5960 M^-1^ cm-^1^. All experiments were conducted in phosphate-buffered saline (PBS) pH 7.2 (Gibco Invitrogen, Carlsbad, CA) unless stated otherwise.

### Preparation of sonicated fibrils

To prepare fibrils, unmodified aSN monomers were dissolved in PBS to 12 mg/ml and incubated horizontally with agitation on a vortex mixer (IKA, Staufen, Germany) for 5 h at 37°C, 900 RPM. Fibrils were pelleted by centrifugation (10 min, 16,000 RCF) and dissolved in 2 ml PBS. Fibril concentration was determined by subtracting the concentration of the soluble aSN after centrifugation from the starting material. Fibrils were sonicated with a probe tip sonicator for 3 times 30 seconds (50% output), stored on ice and used within 30 min.

### Preparation and purification of αSOs

For oligomer preparation, WT aSN monomers were passed through a 100k spin filter to remove preformed aggregates. Unmodified oligomers were prepared by shaking for 5 h at 37°C, 900 RPM, centrifuging to remove fibrils and purified using a Superose 6 column.[35] A 20 mM DHA (Sigma-Aldrich, St. Louis, MO) stock solution was made by adding DHA to PBS while vortexing, followed by 1 min sonication (Branson ultrasonics, Danbury, CT) to generate micelles, and used immediately. HNE-dimethyl acetate (HNE-DMA) (Sigma-Aldrich, St. Louis, MO), which is a stable derivative of HNE, was hydrolysed to HNE according to the manufacturer’s protocol and used within 10 min. To make DHA- and HNE-modified αSOs, 100 μM aSN monomers were incubated with 5 mM DHA in PBS or 2 mM HNE in 30 mM Tris pH 7.2. For both oligomers, mixtures were aggregated in a thermomixer (Eppendorf, Hamburg, Germany) (37°C, 550 RPM) for 24 h. Afterwards the insoluble fraction was pelleted by centrifugation (10min 16.000 RCF at 4°C) and the supernatant was loaded on a Superdex 200 column connected to an Äkta purifier system (GE Healthcare, Uppsala, Sweden) and eluted with PBS at 1.5 ml/min. Oligomer fractions were pooled, sterilized using a 0.22μm filter and stored at 4°C. Samples were used freshly within 24 h unless stated otherwise. Oligomer concentrations were obtained in monomer units using the same extinction coefficient as for monomeric αSN. All experiments described below were performed on purified αSOs. To test the stability of the modified oligomers, 120 μl purified αSOs were injected on the same Superdex 200 column using a 100 μl injection loop.

### Dot blot

WT aSN monomers and purified αSOs were diluted in PBS. 2 **μ**L containing 60 ng sample or buffer was spotted on a nitrocellulose membrane (Cell signalling, Danvers, MA) and air-dried for 30 min at RT. Blots were blocked for 1 h in Odyssey blocking buffer in PBS (LI-COR, Lincoln, NE) and incubated O/N at 4°C with primary antibody diluted in blocking buffer containing 0.1% Tween-20 (Sigma). The following antibody dilutions were used: mouse anti-aSN 211, 1:1000 (sc12767; Santa Cruz biotechnology, Santa Cruz, CA); mouse anti-aggregated aSN 5G4, 1:500 (MABN389, Millipore, Billerica, MA); rabbit anti oligomer A11, 1:500 (AHB0052, Invitrogen, Carlsbad, CA). Subsequent blots were washed 3x 5 min at RT with PBS-Tween 0.1% and incubated for 1 h at RT with the appropriate infrared secondary antibody, IRDye 800CW (LI-COR, Lincoln, NE) diluted 1:5000 in blocking buffer added with 0.1% Tween-20. Blots were washed 3x for 5 min at RT with PBS-Tween 0.1% and 2x for 5 min at RT with PBS, before visualizing using an Odyssey classic infrared imaging system (LI-COR, Lincoln, NE). Images were processed using Image studio lite software (LI-COR, Lincoln, NE, V5.0). The experiment was carried out in triplicate.

### Atomic force microscopy (AFM)

10 μL purified αSOs were added to freshly cleaved mica and allowed to adsorb for 5 min at RT. Unbound protein was gently washed off with Millipore-filtered water (3x 50 μL), dried under a gentle stream of nitrogen and imaged immediately on a MultiMode Nanoscope IIIa microscope (Digital Instruments, USA) equipped with an E-scanner. All measurements were carried out in tapping mode under ambient conditions using single-beam silicon cantilever probes with a resonance frequency of 300 kHz (Olympus, Japan). AFM images were captured with a resolution of 512 samples/line at a scan rate of 1 Hz, with scan sizes of 2x2 μm. Raw data images were processed using Gwyddion software (V2.37) by levelling the data and shifting minimum data values to zero. To determine αSO heights, ~150 αSOs from three different regions were analysed.

### Dynamic light scattering (DLS)

Hydrodynamic radii were acquired using a Zetasizer NANO ZS instrument (Malvern instruments, Worcestershire, UK). Samples were equilibrated to RT and scanned in a disposable solvent-resistant micro cuvette (Malvern instruments, Worcestershire, UK) immediately after αSO purification. For each sample, 16 scans with 14 sub-runs of each 10 seconds were acquired and averaged using the Zetasizer software (V7.10).

### Circular dichroism spectroscopy (CD)

Far-UV wavelength spectra of WT aSN monomer, WT sonicated fibrils and purified αSOs, were recorded from 250 to 190 nm, using a 1 mm quartz cuvette, with a Jasco J-810 spectrophotometer (Jasco Spectroscopic Co. Ltd., Japan). Scans were conducted at 20°C with a step size of 0.2 nm, bandwidth 2 nm, and scan speed of 50 nm/min. Five spectra were averaged for each sample, the buffer spectrum was subtracted, and graphs were smoothed using a 2^nd^ order polynomial and a smoothing window of 10 points. CD spectra were decomposed using online software (http://bestsel.elte.hu/). [36] Fibrils were sonicated before recording spectra.

### Fourier transform infrared spectroscopy (FTIR)

Spectra were recorded on a Frontier FT-IR/FIR spectrometer (Perkin Elmer, Waltham, MA) equipped with a Universal ATR sampling accessory. After measuring the background signal, a 2 μL sample was dried on the crystal using a gentle stream of nitrogen. 64 interferograms were accumulated at a spectral resolution of 2 cm^-1^ in the range of 1000–4000 cm^-1^ and smoothed using a 2nd order polynomial and a smoothing window of 10 points. For comparison, data were normalized. A second order derivative plot was used to determine absorption maxima. Fibrils were sonicated before recording spectra.

### Transmission electron microscopy

Solutions of DHA-αSOs and HNE-αSOs were snap-frozen in liquid nitrogen with 0.2M sucrose for cryoprotection. Subsequently, samples were thawed and 5 μl of sample was transferred to a 400-mesh carbon-coated, glow-discharged grid for 30s, washed with 2 drops of double distilled water, stained with 1% phosphotungstic acid (pH 6.8) and blotted dry on filter paper. Samples were viewed in a JEM-1010 (JEOL, Tokyo, Japan) microscope operating at 60 kV. Images were obtained with an Olympus KeenViewG2 camera and processed with ImageJ software (NIH, V1.48).

### Primary neuron binding experiments

All animal experiments were approved by the Animal Ethical Review Committee (DEC) of Utrecht University, and performed in compliance with the guidelines for the welfare of experimental animals issued by the Federal Government of The Netherlands. Primary hippocampal cultures were prepared and cultured from embryonic day 18 rat brains (Janvier, France) as previously described, with minor modifications.[37] In brief, neurons were plated on coverslips coated with poly-L-lysine (37.5 μg/ml, Sigma) and laminin (1.25 μg/ml, Sigma) at a density of 100,000/well. Dissociated neurons were cultured in Neurobasal medium (Invitrogen) supplemented with 2% B27, 0.5 mM glutamine, 1% penicillin/streptomycin (all from Gibco Invitrogen, Carlsbad, CA,), and 15.6 μM glutamate (Sigma) and incubated at 37°C + 5% CO_2_. After 21 days *in vitro* (DIV 21), coverslips were incubated for 10 min at 37°C + 5% CO_2_ with 100 μL αSOs diluted in PBS. Excess sample was removed by dipping the coverslips in pre-warmed PBS and cells were preserved by incubating in 4% paraformaldehyde (PFA) (Thermo Scientific) + 4% sucrose (Sigma) for 15 min at RT. After 3x PBS washing steps (5 min at RT), cells were incubated ON at 4°C with primary antibodies diluted in antibody dilution buffer (0.2% BSA, 0.8 M NaCl, 0.5% Triton X-100, 30 mM phosphate buffer, pH 7.4). The next day, coverslips were washed 3x with PBS (5 min at RT) and stained with the appropriate secondary antibodies diluted in antibody dilution buffer. Cells were washed 3x with PBS and 2x with MQ water (5 min at RT), mounted using Vectashield mounting medium (Vector laboratories, Burlingame, CA) and stored at 4°C until imaging with the following antibodies: Primary antibodies: rabbit monoclonal anti-aSN (MJFR1) 1:500 (ab138501), chicken polyclonal anti-microtubule associated protein 2 (MAP2) 1:10.000 (ab5392) (both from Abcam, Cambridge, UK), and guinea pig polyclonal anti-vesicular glutamate transporter 1 (vGluT1) 1:400 (ab5905, Millipore, Billirica, MA). Secondary antibodies: AF568 labelled goat anti rabbit 1:400 (A11011), AF488 labelled goat anti guinea pig 1:1000 (A11073) (both from Thermo Fischer, Waltham, MA), and DyLight 405 labelled donkey anti chicken 1:400 (703475155, Jackson Immunoresearch, West Grove, PA). Z-stack images were taken using a confocal microscope (Zeiss LSM-700, Oberkochen, Germany). Maximum intensity projection (MIP) files were made using the supplied Zen software and processed using ImageJ software (NIH, V1.48). For binding analysis, total MJFR1 signal was calculated as integrated density and corrected for the number of cells per image using the total integrated density signal of MAP2. Co-localisation was calculated as described before.[38] Briefly, after opening MIP files and running the ImageJ ‘Puncta Analyzer’ plugin (written by Bary Wark, available upon request), red (MJFR1) and green (vGluT1) channels were manually thresholded to highlight visible puncta without the introduction of background noise. The plugin provides quantitative data for puncta number in each channel, as well as the number of co-localised puncta, which was used to calculate the percentage of MJFR1 puncta which co-localised with vGluT1 puncta.

### Calcein release assay

Small unilamellar vesicles were prepared by chloroform evaporation of 1,2-dioleoyl-sn-glycero-3-phospho-(1’-rac-glycerol) (DOPG) (Avanti Polar Lipids, Alabaster, AL) and resuspending the lipid film in assay buffer (10 mM Hepes, 150 mM NaCl pH 7.2) containing 50 mM calcein (Sigma). The solution was incubated for 1 h at RT with frequent vortexing followed by 5 cycles of freezing in liquid nitrogen and thawing at 65°C. The suspensions were extruded 11 times through a 100 nm filter using the mini extruder set (Avanti polar lipids, Alabaster, AL). Excess calcein was removed using a PD10 column (GE Healthcare, Uppsala, Sweden) and eluted with assay buffer. Calcein-loaded DOPG vesicles were stored at 4°C and used within 24 h. For the calcein release assay, 10μL 5x diluted calcein-DOPG vesicles in PBS were added to a black fluotrac 96-well plate (Greiner Bio-One, Kremsmünster, Austria). Kinetic RFU measurements (ex 480 nm, em 520 nm, 1 h, 37°C, shaking between reads) were started immediately after adding 90 μL of a solution of αSOs or WT monomers of different concentrations in PBS. 12 such solutions were added simultaneously using a multichannel pipette. αSO concentrations given in units of aSN monomer. 90 μl PBS without sample was added as negative control and with 5 μL 10% triton X-100 as positive control. The percentage of permeabilization was determined after subtracting the negative control and by setting the maximum signal obtained after addition of 0.4% triton X-100 to 100%.

### LTP assays

Sagittal hippocampal slices were obtained from adult (6–8 weeks) C57/Bl6 male mice that were anaesthetized by inhalation of isoflurane before decapitation. The brain was rapidly removed and placed into ice-cold Ringer solution where the slices (thickness: 350 μm) were prepared using a vibratome and then transferred into a holding chamber for at least 90 min: the first 30 min at 35°C, the following 60 min cooled down to room temperature. Afterwards the slices were transferred into a superfusing chamber for extracellular recordings. The flow rate of the solution through the chamber was 6 ml/min. The composition of the Ringer solution was 124 mM NaCl, 3 mM KCl, 26 mM NaHCO_3_, 2 mM CaC_l2_, 1 mM MgSO_4_, 10 mM D-glucose, and 1.25 mM NaH_2_PO_4_. It was bubbled with a 95% O_2_−5% CO_2_ mixture and the final pH was 7.3. The experiments were performed at room temperature. All three forms of αSO (unmodified αSOs, HNE-modified αSOs or DHA-modified αSOs) were applied to the superfusion solution at a final concentration of 30-100nM. Extracellular recordings of field excitatory postsynaptic potentials (fEPSPs) were evoked by stimulation of the Schaffer collateral commissural pathway in the dendritic region of hippocampal CA1 and obtained using glass micropipettes (1–2MΩ) filled with artificial cerebrospinal fluid (ACSF). Steady baseline recordings were made for at least 20 minutes before application of tetanic stimuli. For LTP induction, high-frequency stimulation (HFS) conditioning pulses at 100Hz were delivered. The αSOs were applied via the superfusion solution for 90mins previous to LTP induction. The amplified fEPSPs were processed and the data re-analyzed using the “LTP-program“-software,[39] available from https://www.winltp.com). Measurements of the fEPSP slopes were taken between 20 and 80% of the peak amplitude and the slopes are normalized and presented as % EPSP slope of baseline. Data were analysed by ANOVA.

## Results

### DHA and HNE both lead to high yields of stable oligomers with distinct epitopes

**Fig 1:**
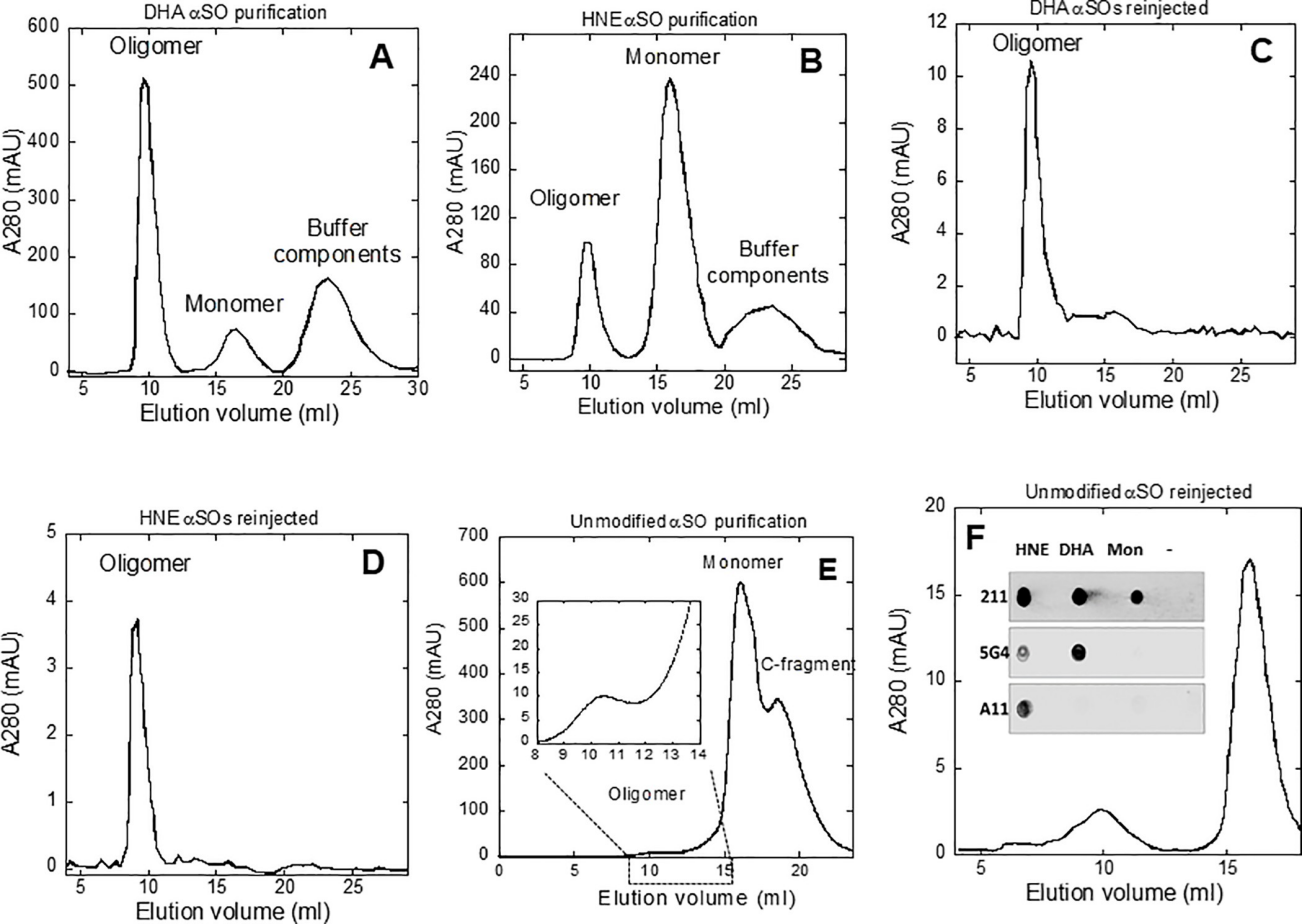
Superdex 200 purification of (A) DHA-αSOs and (B) HNE-αSOs after 24h incubation. Stability of (C) DHA-αSOs and (D) HNE-αSOs was tested by reinjecting the purified oligomers onto the Superdex 200 column, showing that nearly all of the original oligomer was retained. For comparison, Superdex 200 profiles of the preparation of unmofidied αSOs (E) after 5 hrs incubation with shaking and after (F) reinjection of purified oligomers show a much lower yield of oligomer and a much greater degree of dissociation. *Inset*: Dot blot analysis of SEC purified αSOs and aSN monomers.

We first evaluated the level of formation of αSOs in the presence of HNE and DHA compared to unmodified aSN. After 24 h incubation of monomeric aSN at 37°C with a 50-molar excess of DHA or 20-molar excess of HNE, aSN fibrils were pelleted by centrifugation and αSOs were separated from monomers using SEC. Judging by 280 nm peak sizes, incubation with DHA and HNE resulted in high yield (≈60% and 25% respectively) of aSOs (Fig 1A and 1B), both of which eluted as symmetrical peaks, indicating a homogeneous population. HNE-modified oligomers remained completely intact when they were reinjected onto the column while a small amount of DHA-αSO (~20%) appears to dissociate (Fig 1C and 1D). In contrast, unmodified aSN only forms small (1–5%) amounts of oligomer [35,40] (also shown in Fig 1E), most (~80%) of which dissociates upon reinjection (Fig 1F).

We next tested recognition by the conformational specific antibodies A11 and 5G4 to obtain insight into αSOs’ structural features. The polyclonal A11 antibody recognizes structural features of soluble oligomers of amongst others amyloid-β (Aβ), amylin (IAPP) and aSN,[41] and not monomers or mature fibrils of these peptides.[42] While it is still unclear which epitopes are recognized by A11, the motif is conformationally specific and sequence-independent,[43] and is thought to be associated with anti-parallel β-sheet oligomers.[44] Dot blot analysis showed that HNE-αSOs, but not DHA-αSOs and aSN monomers, are recognized by the A11 antibody (Fig 1F). The 5G4 antibody recognizes high molecular weight, β-sheet rich αSOs, with lesser affinity for fibrils and low affinity for monomers.[45] This antibody recognizes DHA-αSOs and to a smaller extent HNE-αSOs. The control antibody 211 recognizes monomeric aSN and also binds to the two αSOs. We conclude that DHA- and HNE-αSOs are recognized by different conformationally specific antibodies, underlining the structural differences between the two species.

### DHA- and HNE-αSOs are rich in β-sheet content

**Fig 2:**
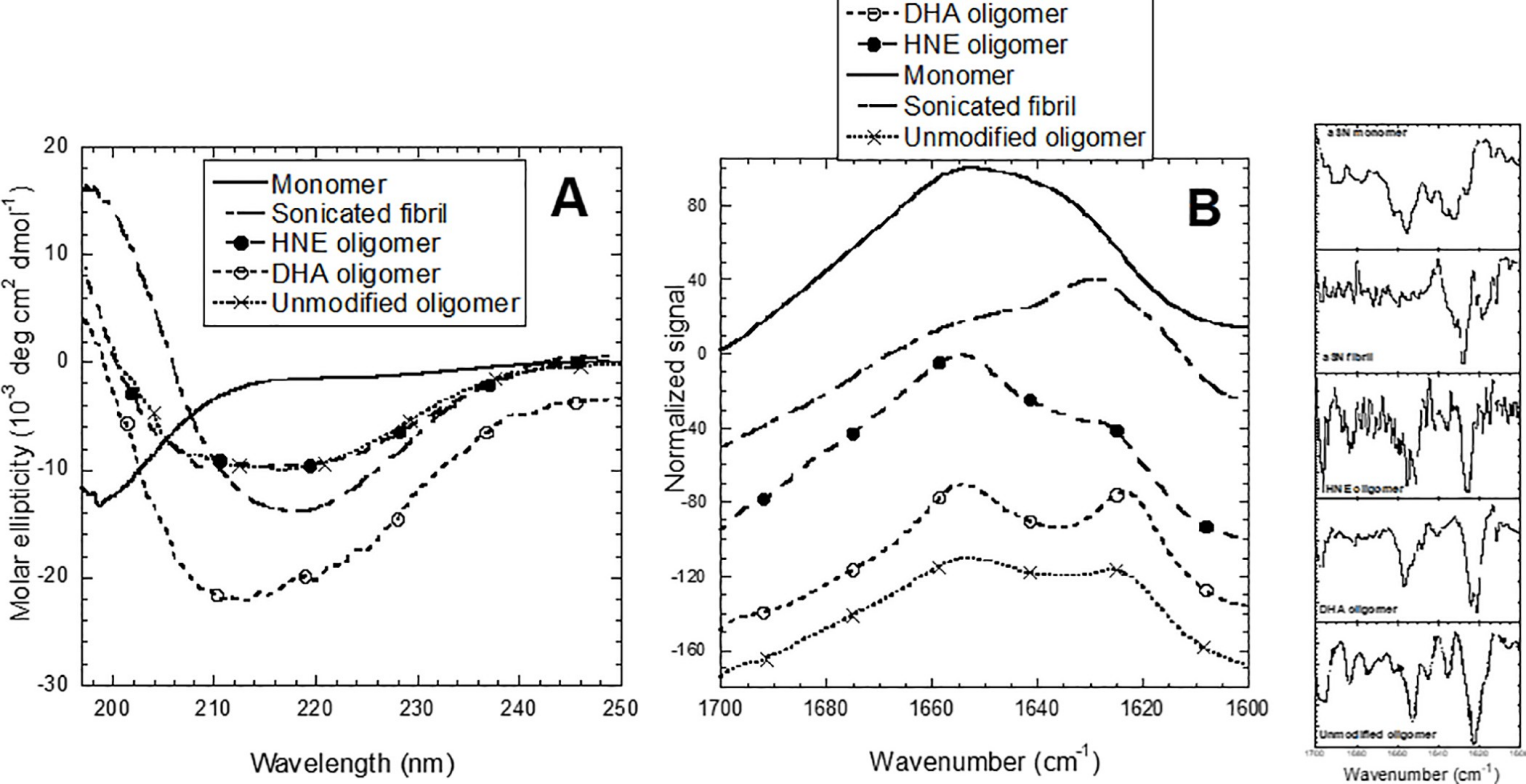
Secondary structure analysis of αSOs. (A) Circular Dichroism and (B) FTIR spectra of DHA αSOs, HNE αSOs, aSN monomer, sonicated aSN fibrils and unmodified αSO. Data for unmodified oligomer taken from.[35] FTIR spectra displaced for clarity.

We next turned to CD and ATR-FTIR to analyse the secondary structure of the two αSOs. Both CD (Fig 2A) and FTIR (Fig 2B) revealed that the two αSOs differed from the monomer and WT fibrils. Whereas CD spectra of the aSN monomers had a minimum around 195 nm, typical for unstructured proteins, WT fibrils showed a typical β-sheet spectrum with a negative band at 218 nm and a positive band around 196 nm. The CD spectra of the αSOs are less distinctly associated with one specific structure. Spectral deconvolution using BeStSel[36] indicates a mixture of random coil (62.3%), α-helical (12.8%) and β-sheet (12.7%) for DHA-αSOs and random coil (45%), α-helical (14.6%) and β-sheet (28.4%) structure for HNE-αSOs. In contrast, the fibrils were predicted to have 47% β-sheet, 8% α-helix and 33% random coil. Additional insight was provided using FTIR spectroscopy. Analysis of secondary derivative spectra revealed a typical random coil structure for the WT monomer (absorption maximum around 1657 cm^-1^). Analysis of the DHA-αSOs revealed a clear β-sheet content. DHA-αSOs also had an absorption maximum in the region 1620–1630 cm^-1^ (typical for amyloid β-sheet),[46] but also featured a maximum around 1695 cm^-1^, characteristic of anti-parallel β-sheet. The FTIR spectra of HNE-αSOs showed a clear peak indicating anti-parallel β-sheets content (absorption maximum around 1620–1630 cm^-1^ with a peak around 1695 cm^-1^), in addition to random coil at 1657 cm^-1^ and a small absorption band around 1683 cm^-1^ assigned to β-turns. The WT fibrils had an absorption maximum around 1620–1630 cm^-1^ typical of β-sheets but no peak around 1695 cm^-1^.

### DHA and HNE-induced oligomers are comparable in size and morphology and are larger than unmodified oligomers

**Fig 3:**
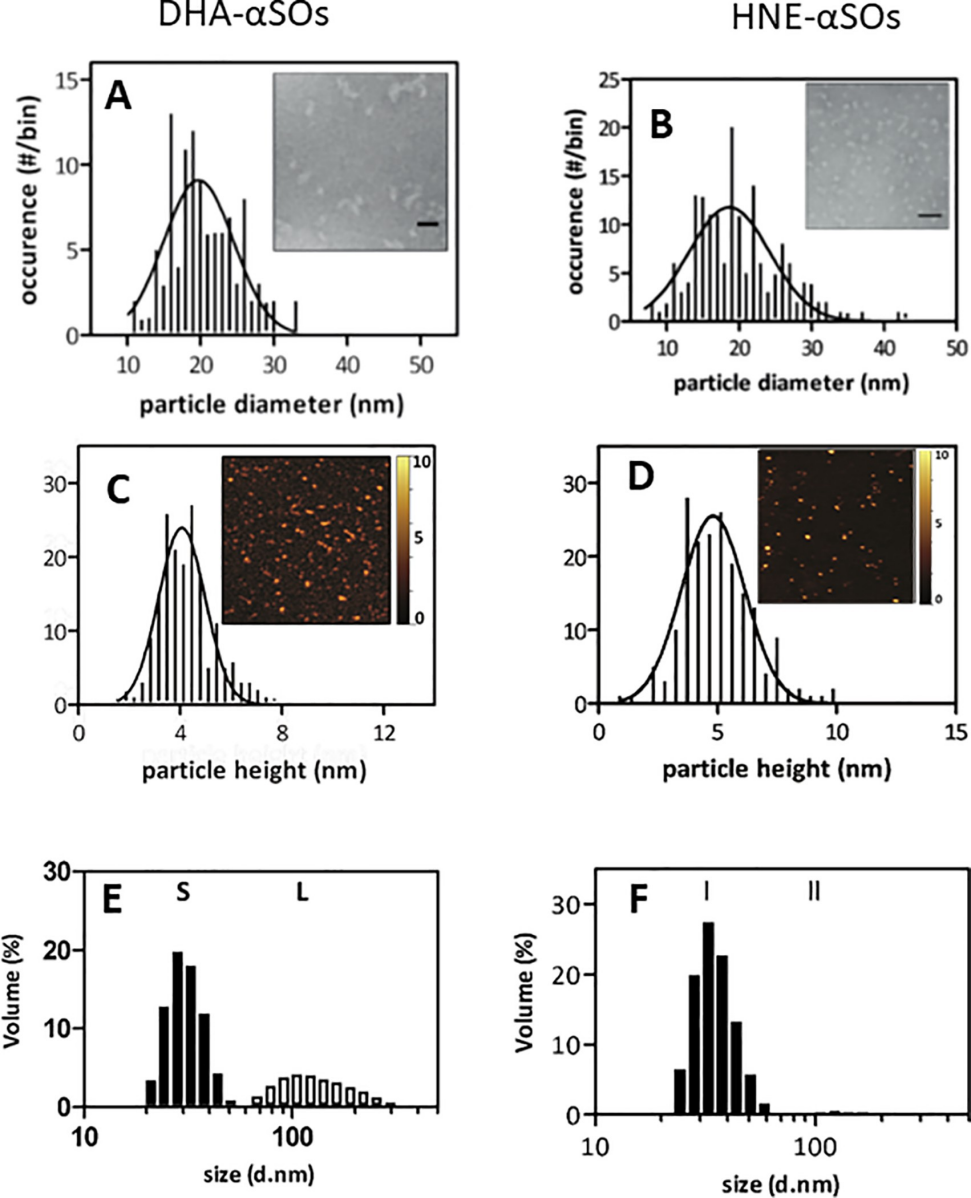
Analysis of the size and shape of DHA-αSOs (left species) and HNE-αSOs (right species). (A and B): Size distribution of TEM species. Insert: TEM image, scale bar is 30 nm. (C and D): Size distribution of AFM images. Insert: AFM image with height scale. (E and F): Size distribution of DLS species.

To obtain details on the overall architecture of the αSOs, we used Transmission Electron Microscopy (TEM) to obtain overall shapes, AFM to measure average height and DLS to determine the hydrodynamic size of the αSOs. TEM images show DHA-αSOs to have spherical morphology and with an average diameter of 20.0±4.6 nm as well as longer curvilinear species which we interpret as stacked spherical αSOs. HNE-αSOs were also spherical with an average diameter of 19.5±6.3 nm (Fig 3B) but had essentially no curvilinear species. AFM images confirmed the spherical shape of the DHA- and HNE-αSOs but the average height under these conditions was 4.4±1.1 and 5.3±1.5 nm (Fig 3C and 3D). In addition we observed smaller spherical species of ~ 1 nm height, likely to be either monomers derived from dissociated oligomers, or small αSOs as reported previously for DHA-αSOs[27], consistent with a small degree of dissociation upon reinjection (Fig 1C). These smaller species were less pronounced for HNE-αSOs, consistent with HNE-αSOs’ greater resistance to dissociation (Fig 1D). The discrepancy in size between AFM and TEM probably reflects a greater extent of collapse on the mica surface coupled with a flattening of the sample which is detected by AFM but not by TEM. Consistent with this, DLS showed that 65.3±8.4% of the volume of the DHA-αSOs consisted of species with a hydrodynamic radius of 37.1±4.9 nm, while the remaining species had an average diameter of 142.2±10.8 nm, attributable to the curvilinear species seen by TEM. 94.2±9.9% of the volume of the HNE-αSOs had a hydrodynamic radius of 37.2±10.3 nm, consistent with the uniformity observed by TEM. Thus, the dominant species of the two classes of oligomers have the same size but differ in the extent to which they form larger and smaller species. Both oligomers, however, were larger than those formed by unmodified aSN, which are spherical or slightly ellipsoid) and with a diameter of 13.9±1.6 nm by TEM [40] height 1–2 nm by AFM[35] and a DLS diameter of 11 nm [35].

### *In vitro* αSOs are able to permeabilize DOPG vesicles

**Fig 4:**
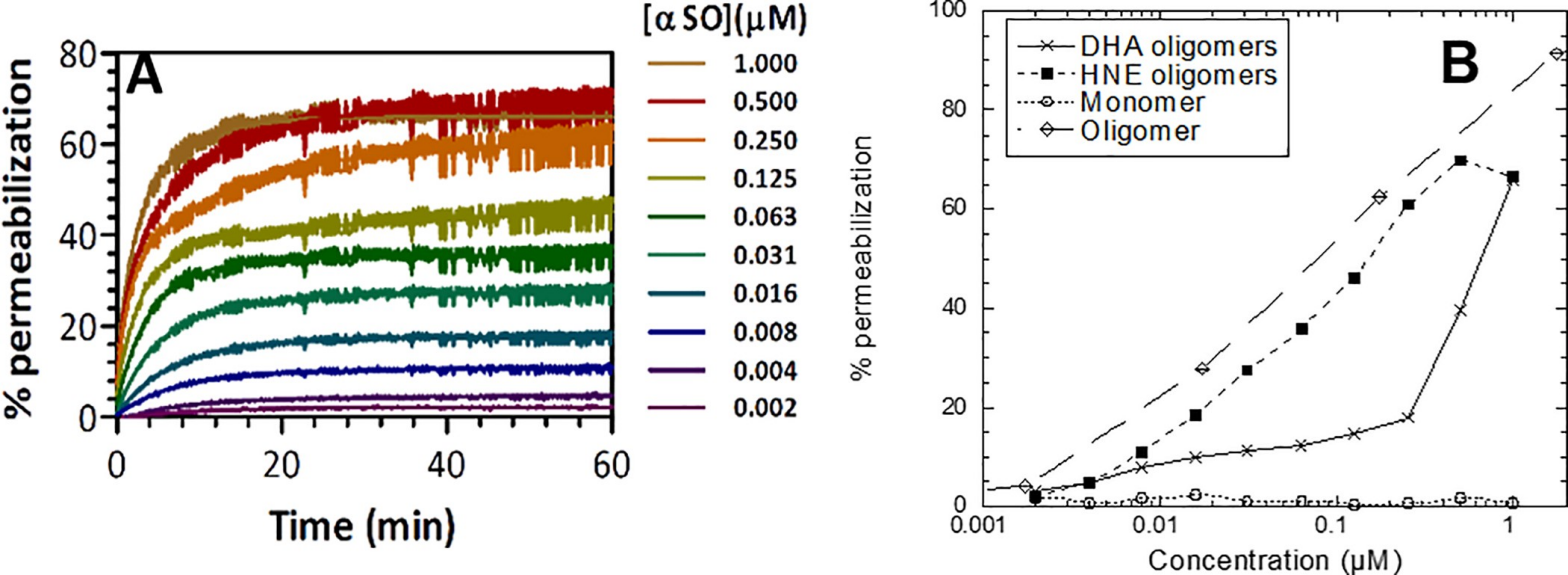
Performance of different αSOs in the calcein release assay. (A) Kinetic RFU (relative fluorescence units) traces of calcein loaded DOPG vesicles after the addition of a dose response of HNE-αSOs in triplicate. Concentrations are based on monomer equivalent. Traces of each triplicate were averaged, subtracted for basal RFU and normalized to 0.4% triton X-100. (B) Degree of permeabilization after 60 min exposure for different aSN species. Data for unmodified oligomer taken from.[35].

αSOs have been shown to induce toxicity, but the exact mechanism is unknown. One of the proposed mechanisms of αSO toxicity is membrane permeabilization. To test the ability of DHA- and HNE-αSOs to permeabilize a model membrane system, different amounts of αSOs were added to calcein loaded DOPG vesicles. The amount of calcein efflux was determined by measuring fluorescence during 60 min incubation with different amounts of WT monomers, DHA- or HNE-αSOs (a typical series of time courses is shown in Fig 4A). Higher concentrations of DHA-αSOs (>0.5 μM) were able to permeabilize DOPG vesicles up to 66.3±0.9% (relative to complete lysis in 0.4% Triton X-100) (Fig 4B). This strong permeabilizing effect was lost when diluting the oligomers to 0.25 μM or lower, where only a moderate effect around 15% leakage compared to Triton X-100 was observed, similar to other reports for DHA-αSO.[27] HNE-αSOs were more membrane-active than DHA-αSOs; they were able to permeabilize DOPG vesicles in a more linear dose response manner (up to 70% for 1 μM of HNE-αSOs) (Fig 4B) and at levels comparable to those of unmodified αSOs.[35] In contrast, similar concentrations of WT monomers were unable to induce calcein efflux (Fig 4B). We conclude that DHA-αSOs and HNE-αSOs permeabilize synthetic vesicles at levels comparable to unmodified αSOs.

### Extracellular added αSOs co-localise with excitatory synapses

**Fig 5:**
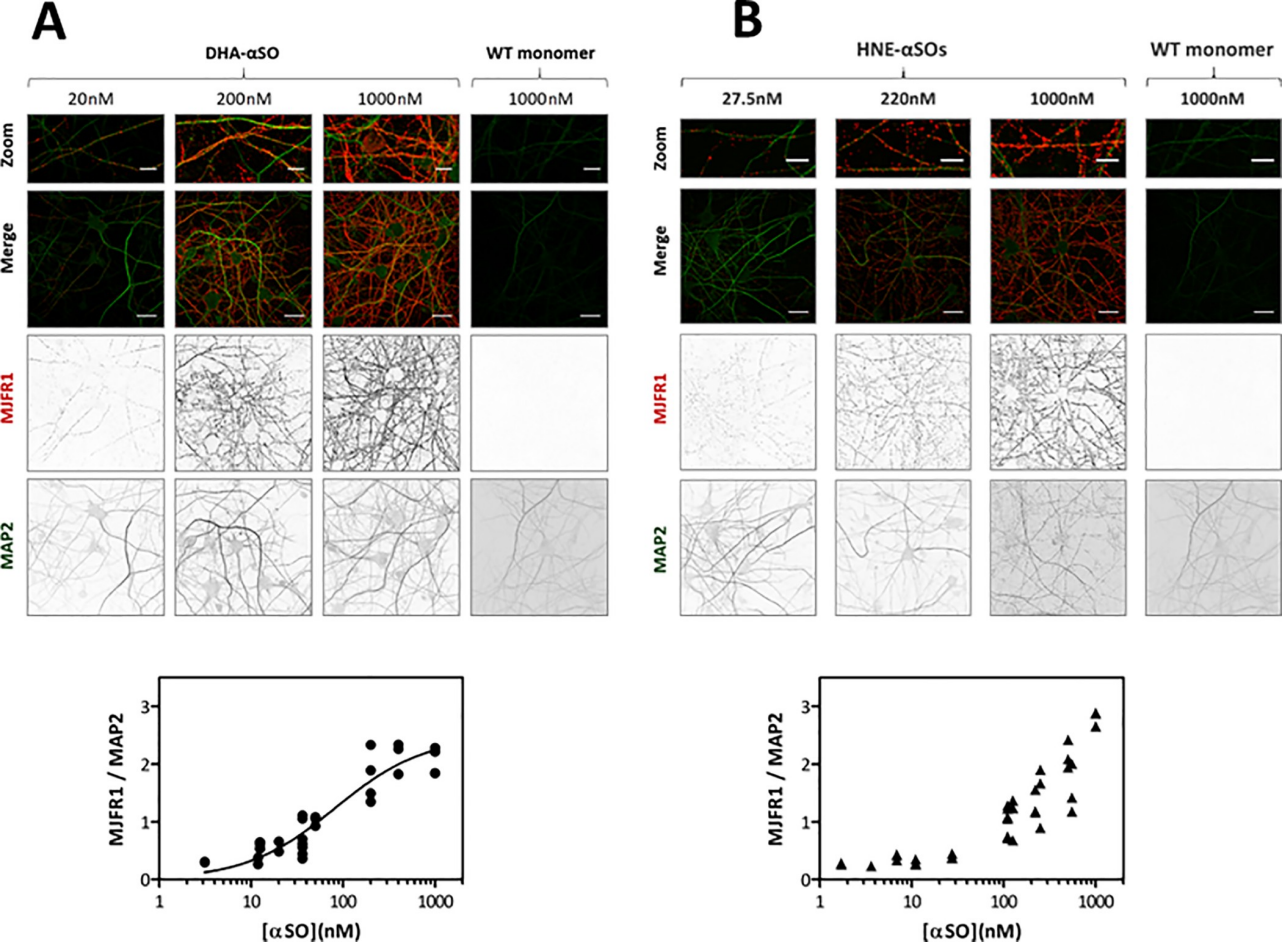
Binding of (A) DHA-αSOs and (B) HNE-αSOs to primary hippocampal neurons. Top: DIV 21 rat hippocampal neurons were incubated for 10 min at 37°C with 1000 nM WT monomers or modified αSOs (3–1000 nM). αSOs (MJFR1, red) and neurites (MAP2, green) were stained followed by confocal imaging. Zoomed image show punctate binding of DHA- or HNE-αSOs to neurites. Scale bars are 30 μm in zoomed image and 5 μm otherwise. Bottom: DHA- or HNE-αSO binding was quantified by measuring the total MJFR1 signal, followed by correction for the amount of cells per image by dividing by the total amount of MAP2 signal. For DHA-αSOs, Kd = 81.85±32.55 nM (Non-linear fit–one site specific with Hill slope).

Synapses are postulated to be the main site of aSN pathology in PD; most of both aSN monomer and aggregated aSN locate at the synapse.[47] Synaptic dysfunction is often observed to precede neurodegeneration and could be caused by either intra- or extracellular αSOs.[48] Fig 5 (top) demonstrates that extracellular DHA- and HNE-αSOs bind primary hippocampal neurons (stained with antibodies against MAP2 which is found in neurites and cell bodies) in a specific punctate pattern. The degree of binding of the DHA-αSOs follows a dose response curve with an estimated K_d_ of 82 ± 33 nM (Fig 5A bottom), although this should not be regarded as the true affinity because the time-dependence of binding was not studied. HNE-αSOs also showed a dose-response curve (Fig 5B bottom), though saturation was less easy to detect, indicating that binding is slightly weaker. No staining was observed upon incubation with 1 μM of WT aSN monomers (Fig 5), indicating that the observed puncta are specifically associated with binding of αSOs. This control image also shows that the MJFR1 antibody is specific for extracellular αSOs of human origin, and does not cross-react with endogenous rat aSN. Even at very low concentrations (3–50 nM), DHA- and HNE-αSOs showed a punctate binding pattern. At higher concentrations the intensity of the MJFR1 signal increased, but binding still occurred in a punctate pattern, indicative of binding to a specific membrane region (*e*.*g*. receptor or other protein), instead of random anionic lipid binding.

**Fig 6:**
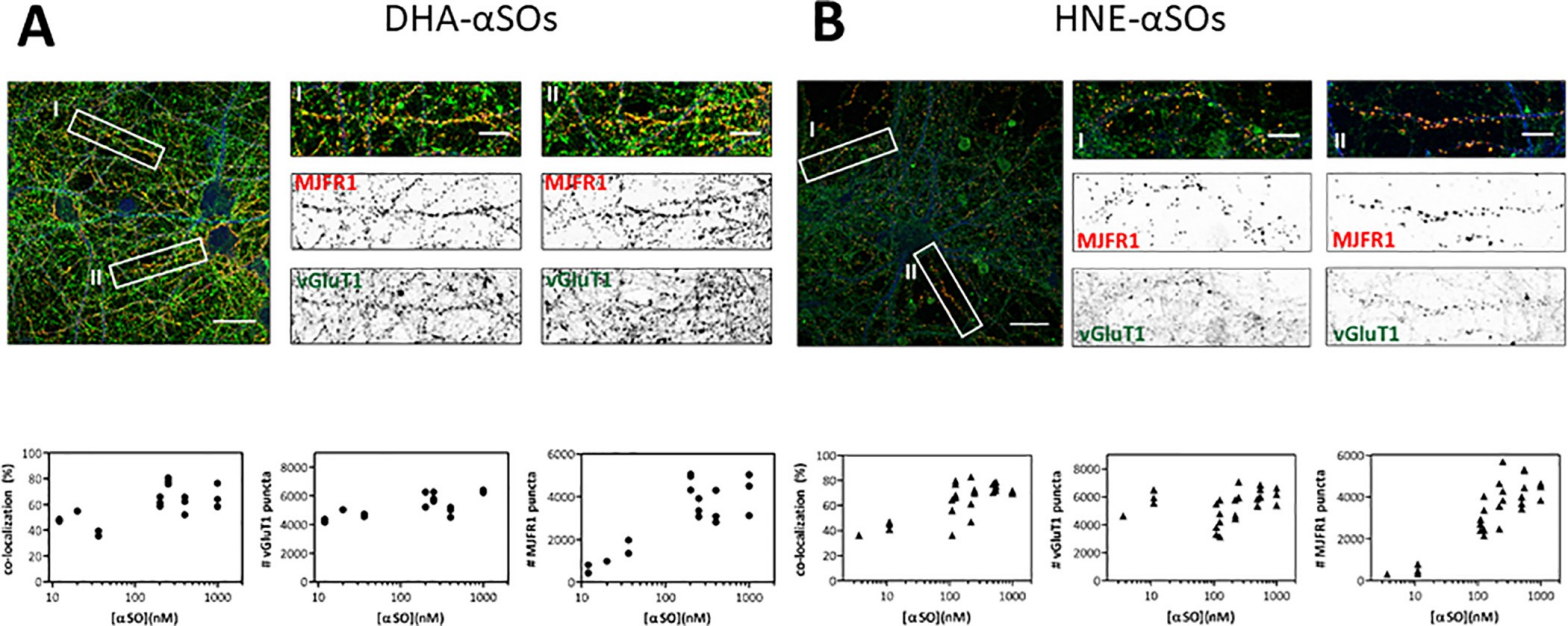
Modified αSOs co-localise with excitatory synapses. (A) DHA-αSOs and (B) HNE-αSOs. (Top) DIV 21 rat hippocampal neurons were incubated for 10 min at 37°C with different amounts of DHA- or HNE-αSOs. Neurites (MAP2, blue), synapses (vGluT1, green) and αSOs (MJFR1, red) were stained followed by confocal imaging. White boxes refer to zoomed images, which show co-localisation of αSOs to synapses (yellow). Scale bars are 30 μm in large image and 10 μm in zoomed images. (Bottom) (i) Co-localisation was quantified using the puncta analyser plugin of ImageJ software. Graph depicts the % of MJFR1 (αSO) puncta which co-localize with vGluT1 puncta. (ii) Quantification of the number of vGluT1 puncta. (D) Quantification of the number of MJFR1 puncta. Each dot represents one analysed image.

To further investigate the punctate binding, neurons were triple stained for glutamatergic synapses (vGluT1), αSOs (MJFR1), and dendrites (MAP2) after 10 min incubation with a range (12–1000 nM) of DHA- and HNE-αSOs (Fig 6 top). Afterwards the amount of co-localisation between MJFR1 and vGluT1 puncta was quantified (Fig 6 bottom). A commonly used method to analyse co-localisation is to measure the amount of yellow (the colour indicating co-localization) per image. However, this method does not discriminate between co-localisation of two puncta and co-localisation with background staining. Since the vGluT1 antibody also shows background staining, the puncta analyser plugin of ImageJ software was used to calculate the percentage of co-localization between puncta. We found that 67 ± 9% of each αSO puncta co-localised with vGluT1 upon incubation with 200–1000 nM DHA-αSOs. This percentage was less (48 ± 10%), but still significant when incubating the neurons with 12–36 nM of DHA-αSOs (Fig 6A). Similarly, 69 ± 11% (n = 22) of each αSOs puncta co-localised with vGluT1 puncta upon incubation with 110–1000 nM HNE-αSOs, which reduced to 42 ± 5% (n = 4), with 3–11 nM HNE-αSOs (Fig 6B). The four images with the lowest concentration and lowest HNE-αSO colocalization were all part of the same batch of neurons. Nonetheless, we do not attribute this to batch variation. All neurons had a similar healthy morphology, based on MAP2 staining, and a similar amount of vGluT1 puncta per image as the other experiments (Fig 6B). We consider unspecific binding to be an unlikely reason for increased co-localisation at higher HNE-αSO concentrations. Although the number of αSO puncta increases upon incubation with 110–1000 nM of HNE-αSOs (Fig 6Biii), the amount of co-localisation stays similar for those concentrations (Fig 5Bi). Rather, we attribute the lack of detection to the small size of the αSO puncta at low HNE-αSO concentrations. Puncta were already small when incubated with 27.5 nM of HNE-αSOs (Fig 6B), they were even smaller when using 3–11 nM HNE-αSOs (images not shown) and hardly exceeded background staining of the MJFR1 antibody. Overall there is no clear αSO concentration dependence in αSO/vGluT1 co-localization, indicating that increasing αSO concentration increases the intensity of existing puncta rather than causing the occupancy of new binding locations due to e.g. unspecific membrane binding. We confirmed binding of αSOs to hippocampal synapses by showing colocalization of αSOs and the post-synaptic marker PSD95 (data not shown). Extracellular DHA- and HNE- αSOs thus show a strong co-localisation with glutamatergic (*i*.*e*. excitatory) synapses independent of the DHA-αSO concentration.

### Different αSO species all negatively affect LTP induction in the CA1 region of the hippocampus

**Fig 7:**
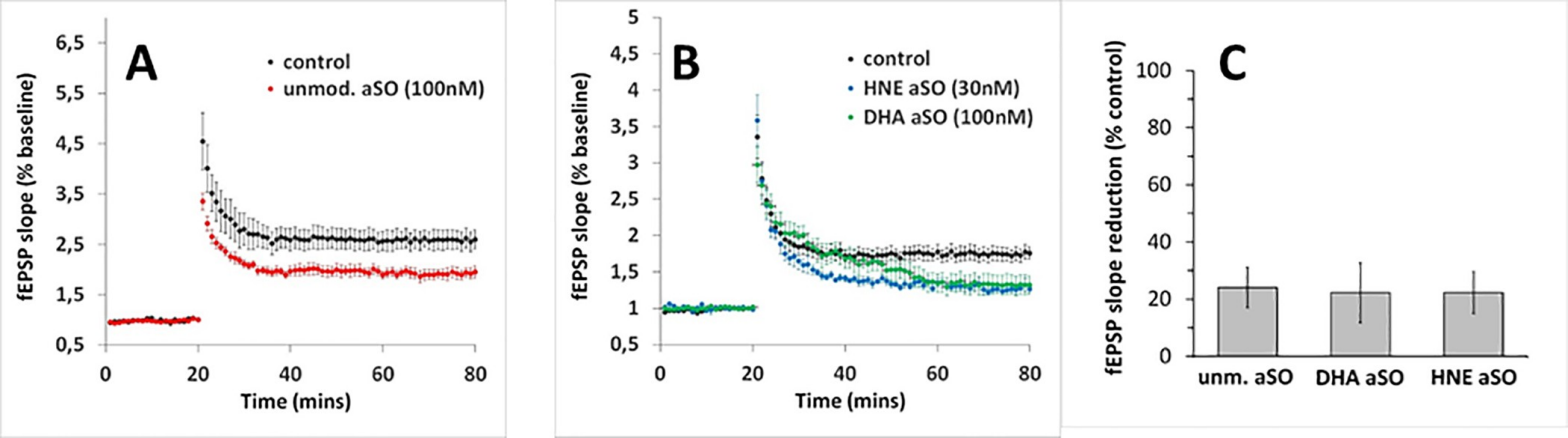
LTP assays with different αSOs. Different αSOs species impair LTP induction in murine hippocampal slices. Prior to high-frequency stimulation (HFS), αSO was applied for 90 min and the responses were measured for the next 60 min. (A) Unmodified αSO (100nM, n = 4, **(B)** DHA-modified αSO (100 nM, n = 7, **B**) and HNE-modified αSO (30 nM, n = 5). Control LTP was averaged for HNE-modified and DHA-modified αSOs (n = 12). (C) The LTP reduction in the presence of the respective αSOs species shown in percent of the fEPSP slope values averaged from the last 10 min of the recordings. The values are displayed as mean ± S.E.M. B2.

Finally we evaluated the potency of the different αSO species in antagonising long term potentiation LTP after tetanic stimulation of the Schaffer collateral. ANOVA analysis revealed that all three tested αSOs, when applied for 90 minutes, significantly antagonized LTP compared to control (F(2;21) = 5263, p<0,0001). In Fig 7, control LTP has been set to 100% to indicate more comprehensively the synaptotoxic effects of αSO species against LTP. Due to limited supplies, only 30 nM HNE αSOs were used as opposed to 100 nM for the other αSO species. Compared to control, all different αSO species decreased LTP induction to the same extent (ca. 30%).

## Discussion

It is generally accepted that αSOs play a key role in PD pathology, but there is no consensus on oligomer size and shape. Since we have little structural information on αSOs in PD patients, it is difficult to predict which *in vitro* generated species, if any, is most relevant for PD pathology. There is no reason to assume that a single species is responsible for all the detrimental effects attributed to αSOs. PD is a complex disease with a rather heterogeneous clinical representation. Each of the associated symptoms may be more or less prominent, or even absent in an individual patient.[49] It is therefore likely that the different αSO species each have their own distinct toxic mechanism, possibly in combination with common features, which in combination lead to PD.

### DHA- and HNE induce high yield oligomers with increased stability

Here we generated *in vitro* αSOs in the presence of DHA or HNE, based on published protocols. We examined the purified αSOs with regards to their structural, morphological, biophysical and functional properties after removing fibrils, monomers and excess excipients. One of the most remarkable effects of the addition of DHA or HNE to a solution of aSN monomer is the substantial increase in oligomer yield compared to the unmodified αSOs. Further, these chemical modifications confer increased stability against dilution, which means that the oligomeric species remain essentially intact and results are unlikely to be significantly perturbed by the presence of dissociated monomers, in contrast to unmodified αSOs. Where possible, we have compared the results of the DHA and HNE-αSOs with previously published results on unmodified αSOs, or by including them as a control to the experiment as summarized in **Table 1**.

**Table 1:** Structural properties of different *in vitro* generated αSOs.

	DHA-αSO	HNE-αSO	Unmodified αSO
**Protocol**	100μM aSN + 5mM DHA	100μM aSN + 2mM HNE	830μM aSN[35,40]
**Purification**	SEC Superdex 200	SEC Superdex 200	SEC Superose 6[35,40]
**Yield oligomers**	60%	20%	1–5%[69] (and this study)
**Size**	1 nm + 4.4 nm (AFM); 37.1 nm (DLS)	5.3 nm (AFM); 37.2 nm (DLS)	1–2 nm (AFM);[35] 4–10.3 nm, 30 monomers (SAXS)[35]
**Morphology**	Spherical (AFM)	Spherical (AFM)	Spherical (TEM)[35] Disc (AFM)[35] Prolate ellipsoid, compact core, diffuse rim (SAXS)[35]
**Secondary structure**	Mixed β-sheet / random coil (CD, FTIR)	Mixed anti-parallel β-sheet/random coil (CD, FTIR)	Mixed β-sheet/random coil (FTIR)[40]
**Antibody recognition**	5G4	A11, 5G4	A1^10^
**Synaptic binding**	Punctate (*K*_d_ of 81.85±32.55 nM)	Punctate	Not tested here
**Co-localisation with vGlut1**	66.6±9.1% of αSO puncta co-localized with vGluT1 (0 2–1μM αSO)	68.9±10.9% of αSO puncta co-localized with vGluT1 (0 11–1μM αSO)	Not tested here
**LTP inhibition**	Yes	Yes	Yes

### DHA- and HNE-αSOs are spherical like unmodified αSOs, but have their own unique structural characteristics

There was a clear variance in size between the species. Whereas DHA- and HNE αSOs are comparable in size to each other as determined by DLS/TEM (37.1/20.0 and 37.2/19.5 nm respectively) and AFM (4.4 and 5.3 nm respectively), they appear larger compared to unmodified αSOs (1–2 nm height as determined with AFM).[35,50] Proteins with a dynamic structure, such as αSOs, are known to collapse when dried on mica.[35] Lipid-modified αSOs may be less affected by this effect in comparison to unmodified αSOs. The difference in height as determined with AFM could thereby indicate a difference in protein rigidity instead of a difference in size. Furthermore, we observed a clear difference in recognition by the anti-aggregated aSN (5G4) antibody and the anti-oligomer (A11) antibody between the species, which can give insight into the structure of αSOs. While both HNE-αSOs and unmodified αSOs[10,41,51] are recognized by A11, DHA-αSOs are recognized by 5G4 instead. The recognition of HNE-αSOs by A11 is in accordance with an earlier report which linked the recognition of A11 with the presence of anti-parallel β-sheet containing oligomers,[44] which were detected using ATR-FTIR. This is, however, in contrast with an earlier report showing that HNE-αSOs are A11 negative [31] and may reflect batch-to-batch variation. Yet, this group reported that their HNE-αSOs had a curved fibrillary morphology and are thereby distinct from our spherical species.[31]

Despite their obvious structural differences, both modified αSOs species shared resemblance with each other and unmodified αSOs. Toxic αSOs are reported to be spherical[35,40,41,50–54] and contain anti-parallel β-sheets,[51,55] and this is also what we observed for DHA- and HNE-αSOs. Both species are a mixture of β-sheet with unordered structures and contain a small amount of α-helical content. DHA αSOs, however, have a stronger general β-sheet character while HNE αSOs have a more pronounced anti-parallel β-sheet conformation. Our result support the previously reported prominent β-sheet character of HNE αSOs, in which also an anti-parallel β-sheet peak in FTIR was observed.[31] It should also be mentioned that one other group showed CD spectra of HNE-αSOs comparable to the unstructured monomer.[29] DHA αSOs, on the other hand, have been shown to be mainly α-helical.[56] However, this α-helical structure appeared immediately after adding DHA and became less pronounced after prolonged exposure to DHA. In addition, α-helical content is reduced after removal of excess DHA,[56] so the initial structure may be ascribed to the well-known tendency of anionic amphiphiles to induce α-helical structure in monomeric proteins when present in excess.[57] Our result indicate a clear β-sheet character and spherical morphology for purified DHA-αSOs, perhaps thanks to the thorough removal of excess DHA and purification of the oligomeric species to remove contaminating monomers.

We conclude that DHA- HNE- and unmodified αSOs are structurally intermediate between the structure of monomer and fibrils but differ from each other on a structural level. The question remains how these structural alterations relate to functional differences. As previously stated, the toxicity of αSOs is strongly dependent on the structural properties of the αSO, where secondary structure is believed to be more important than size.[7,58] Although an all-atom structure of αSO is not available, it is generally believed that it has a high β-sheet content and significant exposure of hydrophobic patches.[7,59,60] As a first step to understanding the oligomers’ functional properties, we tested their ability to permeabilize DPG vesicles and found that HNE-αSOs were almost as active as unmodified αSOs, while DHA-αSOs showed slightly lower activity levels. DHA-αSOs also showed a higher content of α-helicity, and this prompts the speculation that more α-helical-rich structures as found in DHA-αSOs insert into membranes in a less disruptive manner than β-sheet rich structures such as found in both HNE-αSOs and unmodified αSOs. The helices are unlikely to integrate fully into the membrane like transmembrane helices due to their low hydrophobicity compared to *bona fide* membrane proteins; rather, they may bind more superficially to the membrane surface as amphipathic helices, similar to monomeric αSN.[60] In contrast, β-strand structures can insert into the membrane despite having alternating hydrophobic-hydrophilic patterns as seen in outer membrane proteins[61]; a similar pattern might be occurring for HNE-αSOs and unmodified αSOs although this obviously needs to be investigated in greater structural detail.

### Synaptotoxicity as a common toxic mechanism of spherical αSOs

Currently little is known of the mechanisms of αSO toxicity.[16] One important postulated toxic mechanism for *in vitro* generated αSOs is synaptotoxicity. lt has been suggested that αSO toxicity starts within synapses,[47] in line with the observation that synaptic damage precedes neurodegeneration.[61] Synapses are crucial for all aspects of central nervous system function, where they form the basis of chemical communication between neurons.[62] Unmodified αSOs have been shown to impair synaptic functioning by decreasing neuronal excitability,[10] decreasing synaptic firing,[11] inducing a loss of synaptic markers,[61] and impairing long term potentiation (LTP), the latter being a molecular process underlying synaptic plasticity important amongst others for learning and memory.[12] We have shown that DHA- and HNE-αSOs, in contrast to aSN monomers, bind to hippocampal neurons in a dose response fashion, showing a punctate binding pattern. Moreover, we observe a high level of co-localisation with the glutamatergic synapse marker vGluT1. This indicates that αSOs bind to synapses that release the neurotransmitter glutamate, which is the main excitatory neurotransmitter in the brain, and is able to activate for example AMPA and NMDA receptors located at the postsynaptic membrane.[63] A single neuron, and even a single synapse, is able to release several types of neurotransmitters.[64] For example, dopaminergic neurons also release glutamate, and thereby αSO co-localisation with glutamatergic synapses could be relevant for PD pathology. However, there are three distinct vGluT subtypes, and where hippocampal neurons mainly express vGluT1, neurons in the *substantia nigra*, in both human and rodent brain, mainly express vGluT3.[65] It is reasonable to assume that αSOs also co-localise with glutamatergic synapses on dopaminergic neurons. As the resolution of confocal microscopy is limited, we cannot ascribe the αSO binding to a specific receptor or other moiety present on glutamatergic synapses.

From our synaptic binding data we cannot conclude yet which underlying toxic mechanism(s) is activated upon glutamatergic binding, but one possibility is a loss of corticostriatal synaptic plasticity, in the form of LTP impairment, which has been postulated to play a role in PD,[66] and is supported by our data (Fig 7). Previously HNE-αSOs [33] and unmodified αSOs[12] have been reported to impair LTP, which has been suggested to occur via NMDA receptor activation.[33] Our observation that DHA- and HNE-αSOs co-localize with glutamatergic synapses indicates that unmodified- HNE- and DHA-induced αSOs might share this toxic mechanism.

In summary we show that three biochemically distinct αSO species all have the capability to impair LTP, in contrast to WT aSN monomers. Possibly this toxic property of αSOs is linked to the spherical morphology, β-sheet character and relative small size of the three species. aSN seeding requires large aSN aggregates with an elongated morphology, such as sonicated fibrils,[67,68] while spherical αSOs inhibit,[35] or only stimulate aSN monomer aggregation to a modest extent *in vitro*.[51] This seems reasonable, since elongated species of aSN show a higher resemblance to the morphology of mature aSN fibrils. This theory is further supported by our data showing that HNE-αSOs, with a more pronounced spherical character by AFM and TEM, show a similar depression in LTP at a lower concentration compared to DHA-αSOs. However, a final evaluation of the potency of HNE and DHA aSO against the induction of LTP needs a detailed analysis testing more than one concentration. Nevertheless, our observations imply that fibrillar species and spherical αSOs contribute to PD pathology through distinct mechanisms. Given that synaptic damage precedes neurodegeneration,[61] targeting these spherical αSOs could be a good therapeutic strategy.

### Targeting spherical αSOs as a therapeutic strategy for PD

**Fig 8:**
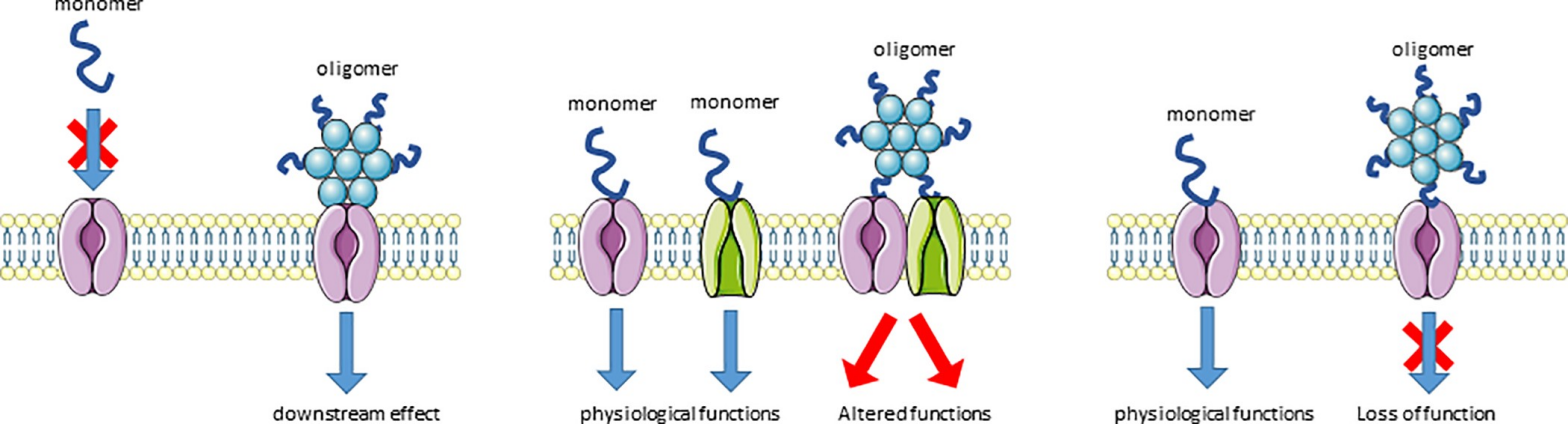
3 different scenarios for oligomer-specific biological effects: (A) Specificity: conformational epitopes unique to αSOs lend unique cellular binding properties to αSO. (B) Avidity: monomeric epitopes that repeat across the oligomeric surface providemultivalency and could drive spatial association of different cellular moieties, causing altered downstream events. (C) Competition: oligomers bind to cellular moieties targeted by monomeric aSN, thereby blocking their normal function. See main text for further details.

Whereas the structural features, morphology, antibody recognition specificities as well as the chemistries underlying the preparation methods of the various investigated αSO species are quite distinct, there is extensive overlap in the αSO’s functional properties as observed in e.g. synaptic binding and LTP experiments. In principle, oligomer-specific biological effects could arise in 3 different ways. (A) Conformational epitopes that are unique to αSOs and do not occur in e.g. monomer or fibrillar aSN, providing αSO with unique cellular binding properties. (B) The presence of (likely flexible) monomeric epitopes that repeat across the oligomeric surface thereby providing multivalency (the avidity effect) which could result in the spatial association of cellular moieties that normally bind monomer, causing altered downstream events as a consequence. (C) A mechanism where oligomers bind to cellular moieties that are normally involved in monomeric aSN binding, thereby blocking normal function of such moieties. This monovalent binding mechanism would predict comparable affinities of monomer and αSO. These scenarios are summarized in Fig 8.

Possibility 3 is less likely because no neuronal binding of exogenously added aSN monomer is observed. The presented results cannot exclude that the oligomers investigated here share unique conformational epitopes (possibility 1) but given the structural differences between the different αSO’s this seems less likely, leaving mechanism 2. This consideration has important implications for therapeutic strategies targeting aggregated aSN. By targeting αSOs directly with using e.g. immunotherapy the first step leading to synaptotoxic events (αSO binding to cells) could be prevented. The development of αSO targeted agents has been hampered by a lack of understanding of αSO toxicity *in vivo*. Many different synthetic αSOs have been described, and it is unclear which species is adequately representative for the *in vivo* situation and, thus, a good model for therapeutic development, rendering the choice of oligomer for development a major risk factor. The realization that multiple oligomeric species have similar biofunctional and synaptotoxic effects is encouraging because it reduces the risks associated with an oligomeric targeted approach: It is likely that therapeutic effects cross over to multiple αSO species, including the elusive pathological αSOs. At the same time, the selection and early development of such therapeutic agents would have to ensure that multiple αSO species are recognized to avoid targeting epitopes that are specific for a given oligomeric subtype and do not occur on (*in vivo*) oligomers.
